# Clinical heterogeneity mapping and machine learning-based prediction of recurrence in gallstone disease: a retrospective cohort study of 9,939 patients

**DOI:** 10.7717/peerj.21699

**Published:** 2026-09-10

**Authors:** Xin Zheng, Yunjun Yan, Ce Bian, Xiaohang Sun, Xue Bai, Xuewei Zhuang, Yanhai Zhang

**Affiliations:** 1 Department of Clinical Laboratory, Shandong Provincial Third Hospital, Shandong University, Jinan, China; 2 Jinan Vocational College of Nursing, Jinan, China; 3 Department of Clinical Laboratory, Jiyang People’s Hospital of Jinan, Jinan, Shandong, China; 4 School of Nursing and Rehabilitation, Shandong University, Jinan, China; 5 Shandong Cancer Hospital and Institute, Shandong First Medical University and Shandong Academy of Medical Sciences, Jinan, China

**Keywords:** Gallstones, Heterogeneity, Recurrence, Risk factors, Machine learning, Prediction model

## Abstract

**Background:**

Gallstone disease constitutes a substantial global health burden, characterized by notable clinical heterogeneity and a complex risk of recurrence. However, large-scale studies systematically characterizing this heterogeneity and translating these findings into clinically applicable prediction tools remain limited. This study sought to systematically elucidate the clinical heterogeneity within a large cohort of gallstone patients, identify independent risk factors for postoperative recurrence, and develop a machine learning-based model for postoperative recurrence prediction.

**Methods:**

We conducted a retrospective analysis of 9,939 patients undergoing gallstone surgery between January 2017 and December 2023 at the Third Hospital of Shandong Province. Clinical data, including demographics, gallstone type, size, comorbidities, and recurrence status, were extracted. Statistical analyses compared subgroups based on sex, age, stone type, complication status, and recurrence. Age- and sex-matched non-recurrence controls were selected in a 1:1 case-control design. Independent recurrence-associated factors were identified using univariate and multivariable logistic regression analyses. A Random Forest model was subsequently constructed using routinely available clinical variables and evaluated in independent training and testing datasets.

**Results:**

Marked clinical heterogeneity was observed across sex, age groups, stone locations, and recurrence status. Distinct associations were identified between specific stone distributions and comorbidity patterns. Multivariable logistic regression identified stone size, diabetes mellitus, venous thrombosis, liver cirrhosis, and malignant tumors as independent factors associated with recurrence. The Random Forest prediction model, incorporating these factors along with sex and age, demonstrated excellent performance with an area under the curve (AUC) of 0.873 on the test set. Variable importance analysis highlighted stone size and cirrhosis as the most influential predictors.

**Conclusions:**

Gallstone disease is characterized by substantial clinical heterogeneity. The identified recurrence-associated factors and machine learning model provide a potential framework for individualized recurrence risk assessment and may facilitate postoperative management following further external and prospective validation.

## Introduction

Gallstone disease constitutes a major global health burden, posing ongoing severe challenges to clinical management (Lammert et al., 2016; Wang et al., 2024). Traditionally, gallstones have often been simplified and treated as a single disease entity based solely on anatomical location. However, accumulating clinical observations reveal extensive variation within the patient population—from sex and age preferences in incidence to the anatomical distribution of stones and vastly different comorbidity profiles (Patel & Jepsen, 2024; Wang et al., 2024). For instance, hepatolithiasis bile duct stones are strongly associated with liver cirrhosis and malignancy (Kim et al., 2015; Motta, Saffioti & Mavroeidis, 2024), whereas cholecystolithiasis more frequently coexists with local gallbladder pathologies such as polyps (Lam et al., 2021). These phenomena strongly suggest that gallstone disease is not a homogeneous condition but rather a “clinical syndrome” driven by diverse underlying pathophysiological mechanisms (Lammert & Miquel, 2008). Systematically elucidating this heterogeneity and understanding its links to clinical outcomes represents the crucial first step in advancing disease cognition from a “one-size-fits-all” approach towards ”precision phenotyping”.

Within this context of complex disease heterogeneity, post-operative recurrence stands as one of the most troublesome and costly clinical problems in gallstone management (Cianci & Restini, 2021; Wu, Xu & Xu, 2021). Although surgical and endoscopic techniques can effectively remove stones, reported long-term recurrence rates remain as high as 24% (Costamagna et al., 2002). Recurrence causes patients to experience pain again and undergo additional procedures, while also imposing a significant economic burden on healthcare systems (Da Costa et al., 2016). Currently, core bottlenecks hindering breakthroughs in recurrence risk management are twofold. First, most existing studies have failed to incorporate the deep-seated heterogeneity of gallstone disease as a framework for predicting recurrence (Li et al., 2022; Wang et al., 2023). Second, traditional prediction models often rely on limited variables and linear methods, insufficiently capturing this complexity. A precise predictive tool grounded in the heterogeneous nature of the disease and capable of integrating multidimensional clinical information using advanced algorithms remains notably absent (Li et al., 2023; Velamazán et al., 2024).

Accumulating evidence has begun to map the microbial landscape associated with gallstones. In a prior analysis of this surgical cohort, we characterized the bile microbiome and identified specific bacterial profiles linked to recurrence (Zheng et al., 2024). This underscores that gallstone disease extends beyond a simple mechanical obstruction. However, a comprehensive understanding of the clinical heterogeneity of the host population—the demographic patterns, stone-type distributions, systemic comorbidity profiles, and their collective association with outcomes—remains largely unexplored. Delineating this clinical heterogeneity is a critical prerequisite for advancing from a one-size-fits-all model to a precision medicine approach.

Although this study was conducted in the same clinical cohort as our previous biliary microbiology analysis (Zheng et al., 2024), the present work addresses a distinct and non-overlapping clinical question. Rather than focusing on biliary microbial characteristics, this study investigates clinical heterogeneity across gallstone subtypes and develops a machine learning-based model for postoperative recurrence prediction using routinely available clinical variables. This design improves clinical interpretability and facilitates practical implementation in routine postoperative management without requiring microbiological sampling or specialized laboratory testing. Therefore, this study aimed to address three progressive scientific questions through a systematic analysis of this near-ten-thousand-patient surgical gallstone cohort: first, to transcend traditional classifications and comprehensively delineate the clinical heterogeneity spectrum regarding demographics, stone types (cholecystolithiasis, extrahepatic bile duct stones (EBD), intrahepatic bile duct stones (IBD) and mixed stones), and comorbidities, thereby identifying distinct clinical phenotypes. Second, within this new phenotyping framework, to precisely identify key independent risk factors associated with postoperative gallstone recurrence. Finally, to integrate all findings to develop and validate a high-performance, machine learning-based prediction model, providing a practical tool for individualized recurrence risk management.

## Materials & Methods

### Study population and data collection

**Figure 1:**
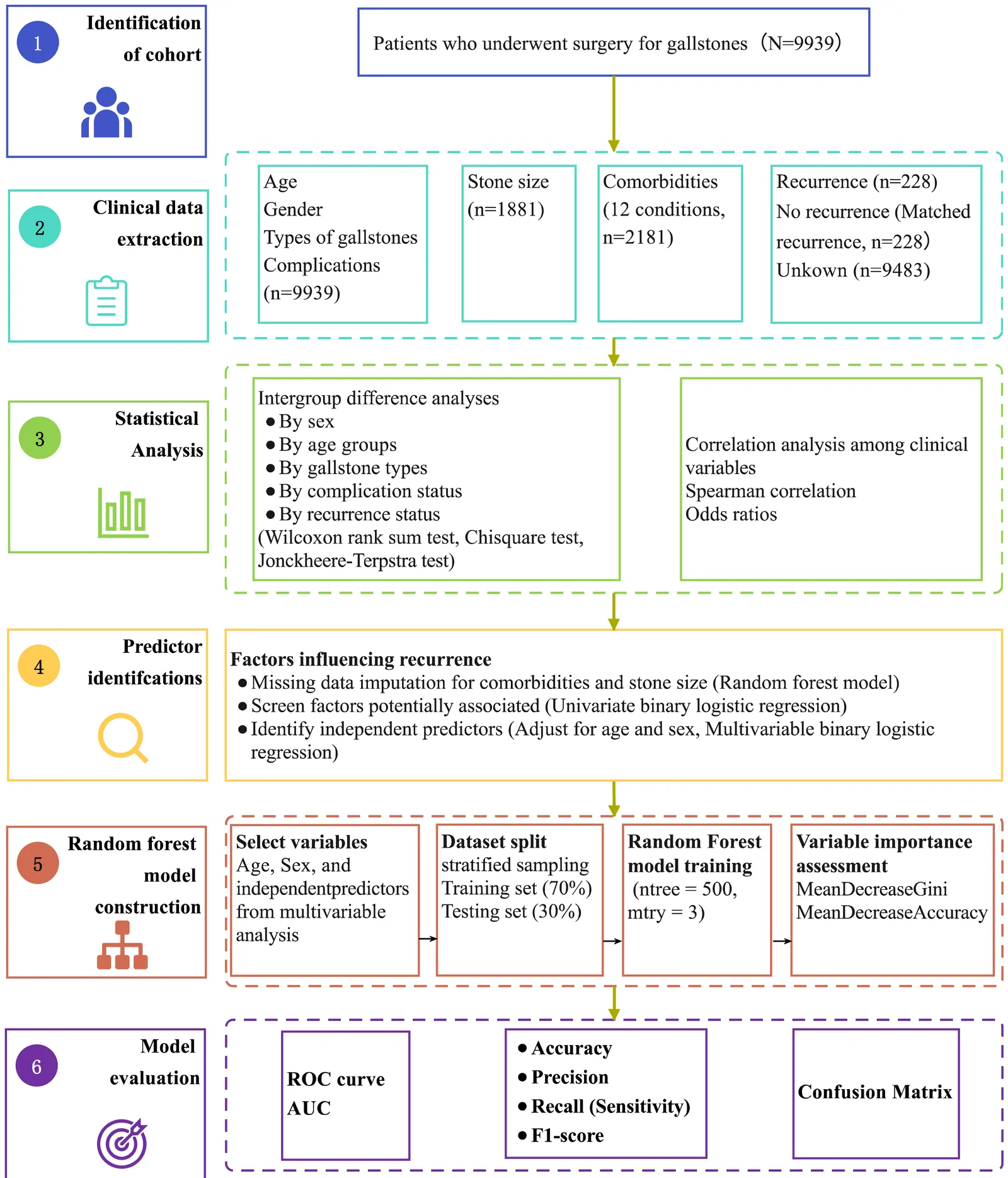
Study design flowchart.

This study utilized the same well-characterized cohort of 9,939 patients who underwent surgery for gallstones at the Third Hospital of Shandong Province between January 2017 and December 2023, as described in our previous work (Zheng et al., 2024). While the prior publication focused on biliary microbiology, the present analysis leverages a non-overlapping set of clinical variables to address distinct hypotheses concerning clinical phenotyping and recurrence prediction. The study was approved by the Shandong Provincial Third Hospital Ethics Committee (Approval No: KYLL-2024064). For participants who underwent follow-up, verbal informed consent was obtained before the telephone interview. For participants whose data were retrospectively extracted from medical records without additional contact, the requirement for written informed consent was waived by the Ethics Committee. Clinical data were retrospectively extracted from electronic medical records, including age, sex, gallstone type (cholecystolithiasis, EBD, IBD, and mixed stones), gallstone size, comorbidities, and recurrence status (Table S1). The overall study design is outlined in Fig. 1.

### Clinical definitions and phenotyping variables

The definitions for key clinical variables were consistent with our prior analysis of this cohort to ensure comparability (Zheng et al., 2024). The following variables formed the basis for clinical phenotyping in this study.

**Gallstone type:** Due to the retrospective nature of the study and the limited availability of detailed imaging and operative records for consistent fine-scale anatomical subclassification (*e.g.*, hilar bile duct involvement), gallstone disease was categorized according to the primary anatomical distribution of stones documented in the electronic medical and radiological records into cholecystolithiasis, intrahepatic bile duct stones, and extrahepatic bile duct stones (Fig. S1). Cholecystolithiasis was defined as stones confined to the gallbladder lumen. Hepatolithiasis/Intrahepatic bile duct stones were defined as stones located within the intrahepatic biliary ductal system inside the liver parenchyma, including segmental and subsegmental intrahepatic bile duct branches. Extrahepatic bile duct stones were defined as stones located within the extrahepatic biliary tract outside the liver, including the common hepatic duct and common bile duct. Patients with simultaneous involvement of multiple anatomical regions were classified as mixed-type stones.

**Complications:** Defined as the occurrence of gallstone-related cholecystitis, cholangitis, or pancreatitis.

**Recurrence:** Recurrence was defined as re-hospitalization at our institution with imaging-confirmed gallstones more than 6 months after the initial surgery, provided that no residual stones were identified on postoperative imaging assessment (Von Schönfels et al., 2013). This definition was intended to minimize misclassification due to retained postoperative stones. Although postoperative imaging evaluation was used to exclude residual stones, the possibility of retained small calculi being misclassified as recurrence cannot be completely eliminated in a retrospective study setting. Because long-term follow-up was incomplete for a substantial proportion of the overall cohort, the present study did not estimate postoperative recurrence incidence. Instead, recurrence analyses were restricted to patients with documented recurrence identified through return visits to our institution.

**Non-recurrence:** For the non-recurrence group, controls were selected using 1:1 age- and sex-matching to recurrence cases from individuals without documented recurrence, who had not been re-hospitalized for gallstone recurrence and had at least two years of follow-up after surgery. However, because follow-up was based on available medical records and patient-reported information rather than routine scheduled imaging surveillance, asymptomatic recurrence may not have been fully excluded.

**Comorbidities:** The analysis included the 12 most prevalent conditions: hypertension, respiratory diseases, coronary heart disease, diabetes, renal cyst, cerebral infarction, fatty liver, venous thrombosis, gallbladder adenomyomatosis, gallbladder polyp, malignant tumor, and liver cirrhosis. (Detailed subcategories for “respiratory diseases” and “malignant tumors” are provided in Supplementary Methods).

**Stone size:** Defined as the longest diameter of the largest stone.

### Statistical analysis

In descriptive statistics, normally distributed continuous variables are presented as mean ± standard deviation (SD), while non-normally distributed variables are reported as median with interquartile range (IQR). Categorical variables are reported as frequencies and percentages.

The Wilcoxon rank-sum test was used to compare non-normally distributed continuous variables between two groups. The Chi-square test was utilized for comparing categorical variables between two groups. For comparisons among ordered categorical variable groups, the Jonckheere-Terpstra test was used for continuous variables, and the Chi-square test followed by post-hoc multiple comparisons was used for categorical variables. Trends in categorical variables across ordered groups were analyzed using the Cochran-Armitage Trend Test.

The Spearman’s correlation coefficient was used for correlation analysis involving ordered categorical or continuous variables. Odds ratios (OR) with 95% confidence intervals (CI) were calculated to analyze relationships between two categorical variables.

### Missing data imputation

In the analysis of factors influencing recurrence, missing data for comorbidity and maximum gallstone diameter were imputed using the missForest package in R, a non-parametric missing value imputation method based on a Random Forest model. The imputed dataset was subsequently used for downstream univariate and multivariable regression analyses as well as Random Forest model development. The out-of-bag (OOB) error rates were used to report the imputation accuracy.

### Analysis of recurrence factors and prediction model construction

Univariate binary logistic regression analysis was first performed to screen for factors potentially influencing recurrence. Variables with *P* < 0.05 in the univariate analysis, along with sex and age as fundamental variables, were included in a multivariable binary logistic regression analysis to identify independent predictors.

A Random Forest model was constructed to predict recurrence. The dataset was split into training and testing sets using stratified sampling (70% for training and 30% for testing) *via* the caret package in R, ensuring a comparable distribution of recurrence and non-recurrence cases between the two sets. The model was built using the randomForest package. Variable importance was assessed by the MeanDecreaseGini and MeanDecreaseAccuracy metrics. The model’s performance was evaluated using the area under the receiver operating characteristic curve (ROC-AUC), accuracy, precision, recall, and F1-score.

All statistical tests were two-sided, with a *P*-value < 0.05 considered significant. Analyses were performed using SPSS^®^ Statistics version 22 (IBM, Armonk, NY, USA) and R version 4.3.2 (R Core Team, 2023).

## Results

### Baseline characteristics of the gallstone cohort

The comprehensive baseline demographic and clinical characteristics of this 9,939-patient gallstone surgery cohort have been detailed previously (Table 1) (Zheng et al., 2024). In summary, the cohort had a slight female predominance (53.30%), a median age of 64 years, and cholecystolithiasis (52.21%) was the most common gallstone type, followed by EBD (40.17%).

**Table 1:** Demographic and clinical characteristics (Zheng et al., 2024).

Characteristic	Designation	Data	Min–max
Sex (*n* = 9,939)	Male	4,640 (46.70)	/
	Female	5,299 (53.30)	/
Age (*n* = 9,939)	/	64 (52, 75)	4–104
Age groups (*n* = 9,939)	<45	1,488 (14.97)	/
	45–59	2,511 (25.26)	/
	60–74	3,420 (34.41)	/
	≥75	2,520 (25.36)	/
Types of gallstones (*n* = 9,939)	Cholecystolithiasis (C)	5,189 (52.21)	/
	Extrahepatic bile duct stones (EBD)	3,992 (40.17)	/
	Intrahepatic bile duct stones (IBD)	504 (5.07)	/
	C+EBD	183 (1.84)	/
	EBD+IBD	62 (0.62)	/
	C+IBD	4 (0.04)	/
	C+EBD+IBD	5 (0.05)	
Stone size (*n* = 1,881)	Maximum stone diameter (mm)	10 (6, 15)	2–60
Complications (*n* = 9,939)	No complications	6,731 (67.72)	/
	With complications	3,208 (32.28)	/
Comorbidities (*n* = 2,181)	Hypertension	918 (42.09)	/
	Respiratory diseases	874 (40.07)	/
	Coronary heart disease	474 (21.73)	/
	Diabetes	436 (19.99)	/
	Renal cyst	312 (14.31)	/
	Cerebral infarction	302 (13.85)	/
	Fatty liver	270 (12.38)	/
	Venous thrombosis	266 (12.20)	/
	Gallbladder adenomyomatosis	251 (11.51)	/
	Gallbladder polyp	211 (9.67)	/
	Malignant tumor	153 (7.02)	/
	Cirrhosis of the liver	90 (4.13)	/
Recurrence status	Recurrence	228 (2.30)	/
	No recurrence (Matched recurrence)	228 (2.30)	/
	Unkown	9,483 (95.4)	/

**Notes.**

Categorical variables were presented as n (%). Normally distributed continuous variables were depicted by Mean ± SD, and nonnormally distributed continuous variables were represented as Q2 (Q1, Q3). Q1: First quartile; Q2: Second quartile; Q3: Third quartile.

Among the 1,881 patients with available data, the median maximum stone diameter was 10 mm (IQR: 6–15 mm), with 92.34% of stones ≤20 mm (Fig. S2A). Comorbidity information was available for 2,181 patients, all of whom had at least one condition. The most prevalent comorbidities were hypertension (42.09%) and respiratory diseases (40.07%), and the number of patients decreased as comorbidity count increased (Fig. S2B). A total of 317 unique comorbidity combinations were identified, with “respiratory diseases alone” (9.31%) and “hypertension alone” (8.02%) being the most frequent (Fig. S2C).

Through review of hospital readmission records, 228 patients were identified as having imaging-confirmed recurrent gallstones. It is important to note that these 228 cases represent only those patients who returned to our hospital for evaluation of recurrent symptoms. Asymptomatic recurrences or patients who sought care at other institutions were not captured, and the true population-level recurrence rate cannot be reliably estimated from this cohort.

### Group differences in clinical characteristics

To delineate the clinical heterogeneity of gallstone disease, we performed a comprehensive comparative analysis across different patient subgroups.

**Figure 2:**
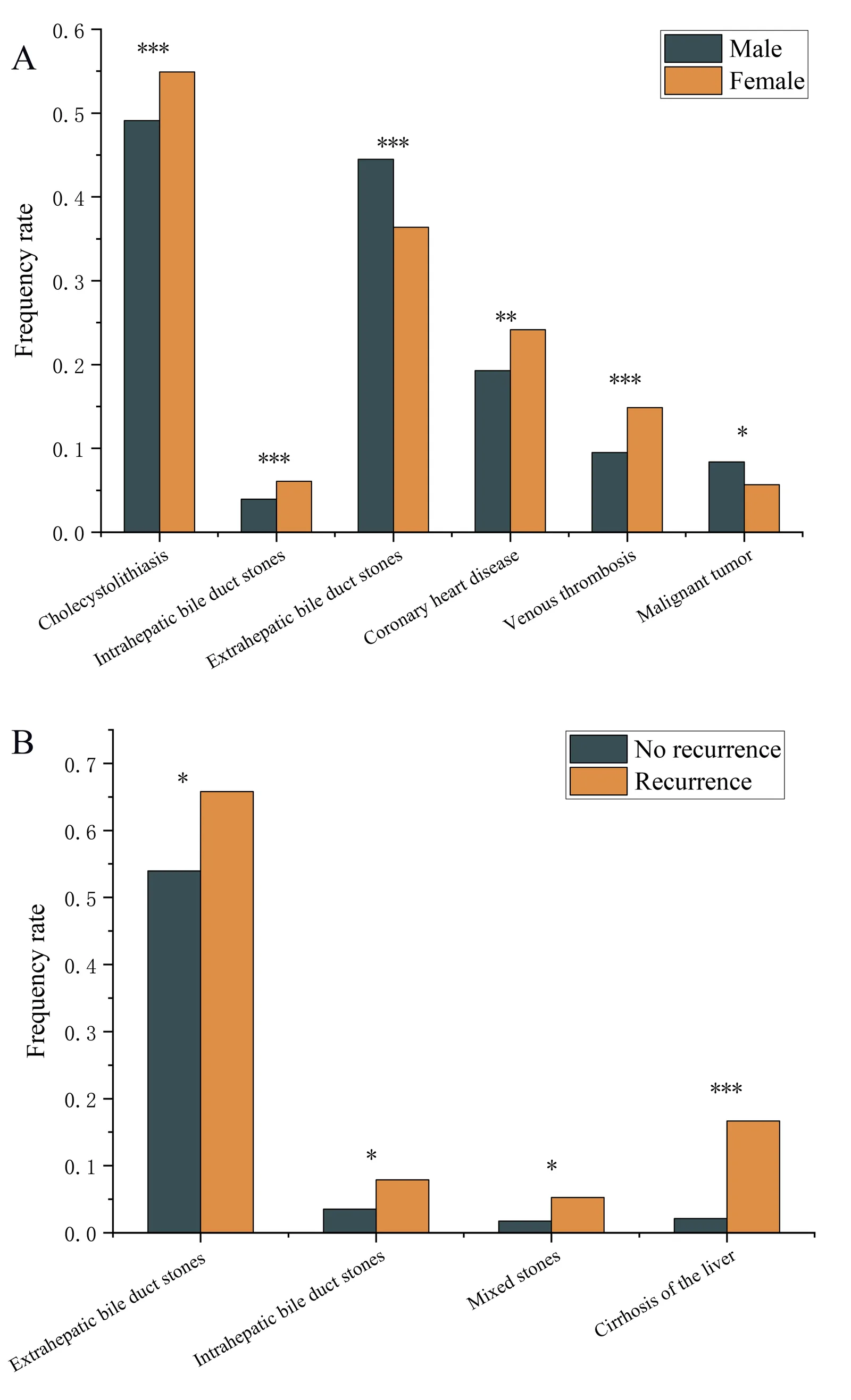
Comparison of clinical characteristics by sex and recurrence status. (A) Comparison of clinical variables between male and female groups. (B) Comparison of clinical variables between recurrence and non-recurrence groups. Only variables with statistically significant between-group differences are presented. Data are shown as proportions. Statistical significance was determined using the Chi-square test. Note: *P < 0.05; **P < 0.01; ***P < 0.001.

**Sex-based differences** (Fig. 2A). Females had higher prevalence of cholecystolithiasis (*P* < 0.001) and IBD stones (*P* < 0.001), whereas EBD stones were more common in males (*P* < 0.001). Coronary heart disease (*P* = 0.005) and venous thrombosis (*P* < 0.001) were more frequent in females, while malignant tumors were more prevalent in males (*P* = 0.013).

**Figure 3:**
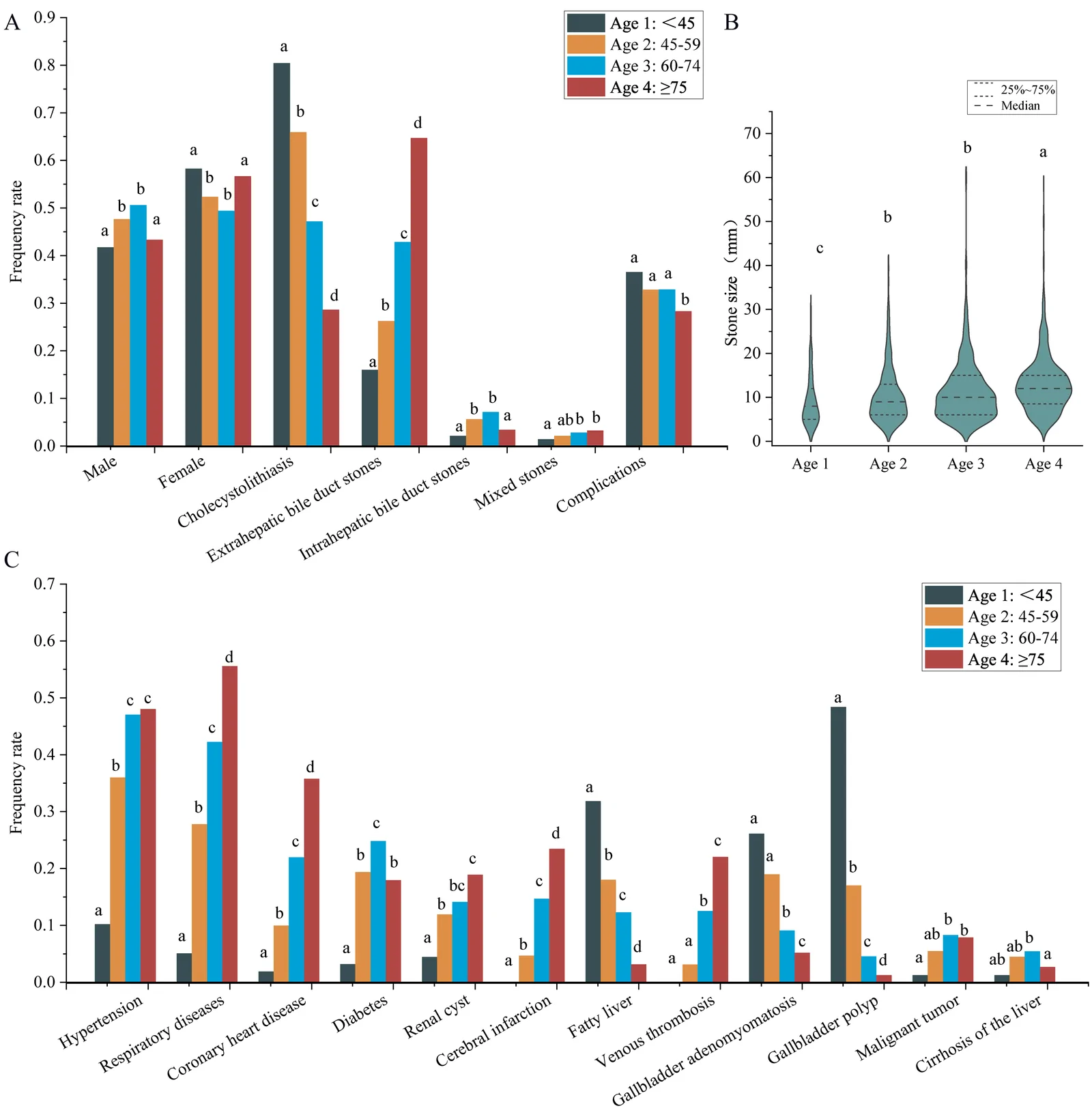
Age-stratified differences in clinical characteristics in the study cohort. The study cohort was stratified into four age groups: <45 years, 45–59 years, 60–74 years, and ≥75 years. (A): sex, types of gallstones, complications; (B): comorbidities; (C): stone size. Only variables with statistically significant differences between groups are shown. Lowercase letters (a, b, c, d) above the bar chart indicates the difference significance; different letters indicate a significant difference between two groups, while the same letter indicates no difference between two groups.

**Age-stratified analysis** (Fig. 3). Patients were categorized into four age groups: <45, 45–59, 60–74, and ≥75 years. The sex ratio differed significantly (*P* < 0.001), with a higher proportion of males in the 45–59 and 60–74 year groups. The prevalence of cholecystolithiasis decreased with advancing age, while that of EBD stones increased (both *P* < 0.001) (Fig. 3A). The prevalence of most comorbidities, including hypertension, respiratory diseases, coronary heart disease, diabetes, renal cysts, and cerebral infarction, increased with age, whereas fatty liver, gallbladder polyps, and gallbladder adenomyomatosis decreased (Fig. 3B). A weak positive correlation was observed between age and stone size (Spearman’s *r* = 0.238, *P* < 0.001) (Fig. 3C).

**Gallstone type-specific profiles** (Fig. S3). The EBD group had the highest proportion of males, while the cholecystolithiasis group had the smallest stone size and the highest complication rate (all *P* < 0.001) (Figs. S3A and S3B). Regarding comorbidities (all except diabetes differed significantly), hypertension, respiratory diseases, and venous thrombosis were more frequent in the EBD and IBD groups. In contrast, gallbladder adenomyomatosis and polyps were more common in the cholecystolithiasis group. Cirrhosis and malignant tumors were significantly more prevalent in the IBD and mixed stones groups (Fig. S3A).

**Complication status** (Fig. S4). Patients without complications were significantly older and had larger stones compared to those with complications (both *P* < 0.001) (Figs. S4A, S4B). EBD, IBD, and mixed stones were more frequent in the no complications group, while cholecystolithiasis was more common in the complications group (all *P* < 0.001). Renal cysts (*P* = 0.005) and malignant tumors (*P* = 0.021) were more prevalent in patients without complications (Fig. S4C).

**Recurrence** ***vs.*** **Non-Recurrence** (Fig. 2B). Compared to the matched non-recurrence group, patients with recurrent gallstones had a significantly higher prevalence of EBD stones (*P* = 0.010), IBD stones (*P* = 0.043), and mixed stones (*P* = 0.042) (Fig. 2B). Furthermore, cirrhosis was significantly more common in the recurrence group (*P* < 0.001).

### Correlation analysis among clinical variables

A weak positive correlation was observed between age and stone size (Spearman’s *r* = 0.255, *P* < 0.001), consistent with the trend identified in the age-stratified analysis. OR analysis revealed a network of significant associations (Fig. S5). Cholecystolithiasis was positively associated with gallbladder polyps (OR = 8.119; 95% CI [5.299–12.441]) but negatively associated with cirrhosis (OR = 0.134; 95% CI [0.073–0.248]) and recurrence (OR = 0.131; 95% CI [0.086–0.199]). Conversely, EBD stones were positively associated with recurrence (OR = 5.149; 95% CI [3.452–7.681]) and negatively associated with gallbladder polyps (OR = 0.15; 95% CI [0.097–0.232]). IBD stones were strongly associated with cirrhosis (OR = 6.689; 95% CI [3.930–11.384]). Furthermore, mixed stones were positively associated with both malignant tumors (OR = 6.74; 95% CI [1.669–27.218]) and cirrhosis (OR = 11.983; 95% CI [2.948–48.711]).

### Factors influencing gallstone recurrence

**Figure 4:**
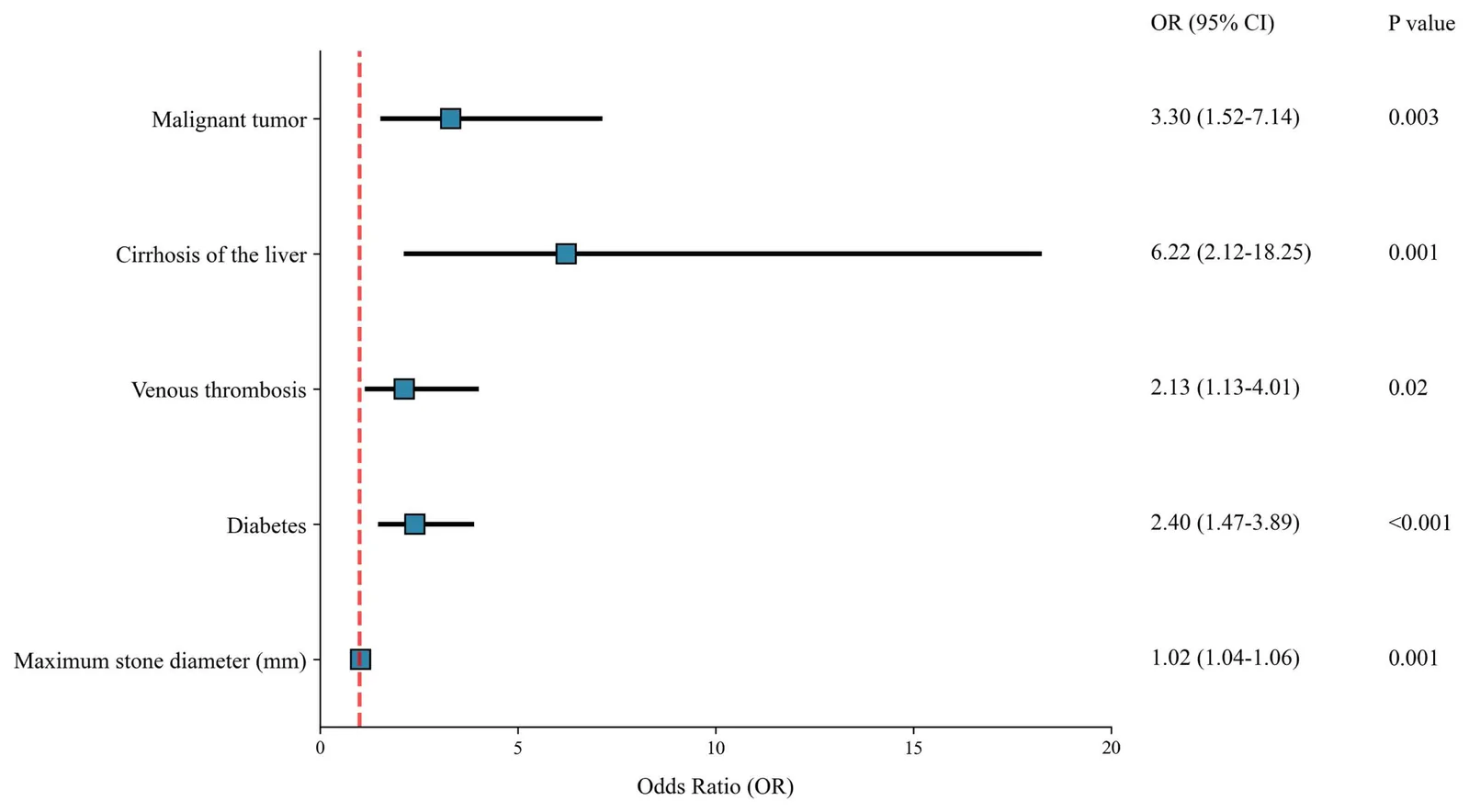
Forest plot of multivariable binary logistic regression analysis identifying independent predictors of gallstone recurrence. ORs and 95% CIs are shown for each significant predictor. Stone size is presented as OR per one mm increase.

As the time to recurrence can be subject to patient-dependent factors, this study focused on the binary outcome of recurrence (presence or absence). After imputing missing data for comorbidities and stone size using a Random Forest model, univariate binary logistic regression analysis identified several variables associated with recurrence (Table S2). These variables were subsequently included in a multivariable binary logistic regression model, which demonstrated a good fit with the observed data (Hosmer and Lemeshow Test, *P* = 0.162). The analysis identified five independent predictors of recurrence (Fig. 4): stone size (OR = 1.037 per one mm increase; 95% CI [1.015–1.060]), diabetes (OR = 2.396; 95% CI [1.474–3.893]), venous thrombosis (OR = 2.126; 95% CI [1.126–4.013]), cirrhosis of the liver (OR = 6.223; 95% CI [2.122–18.249]), and malignant tumor (OR = 3.295; 95% CI [1.521–7.137]).

### Development and performance of a recurrence prediction model

**Figure 5:**
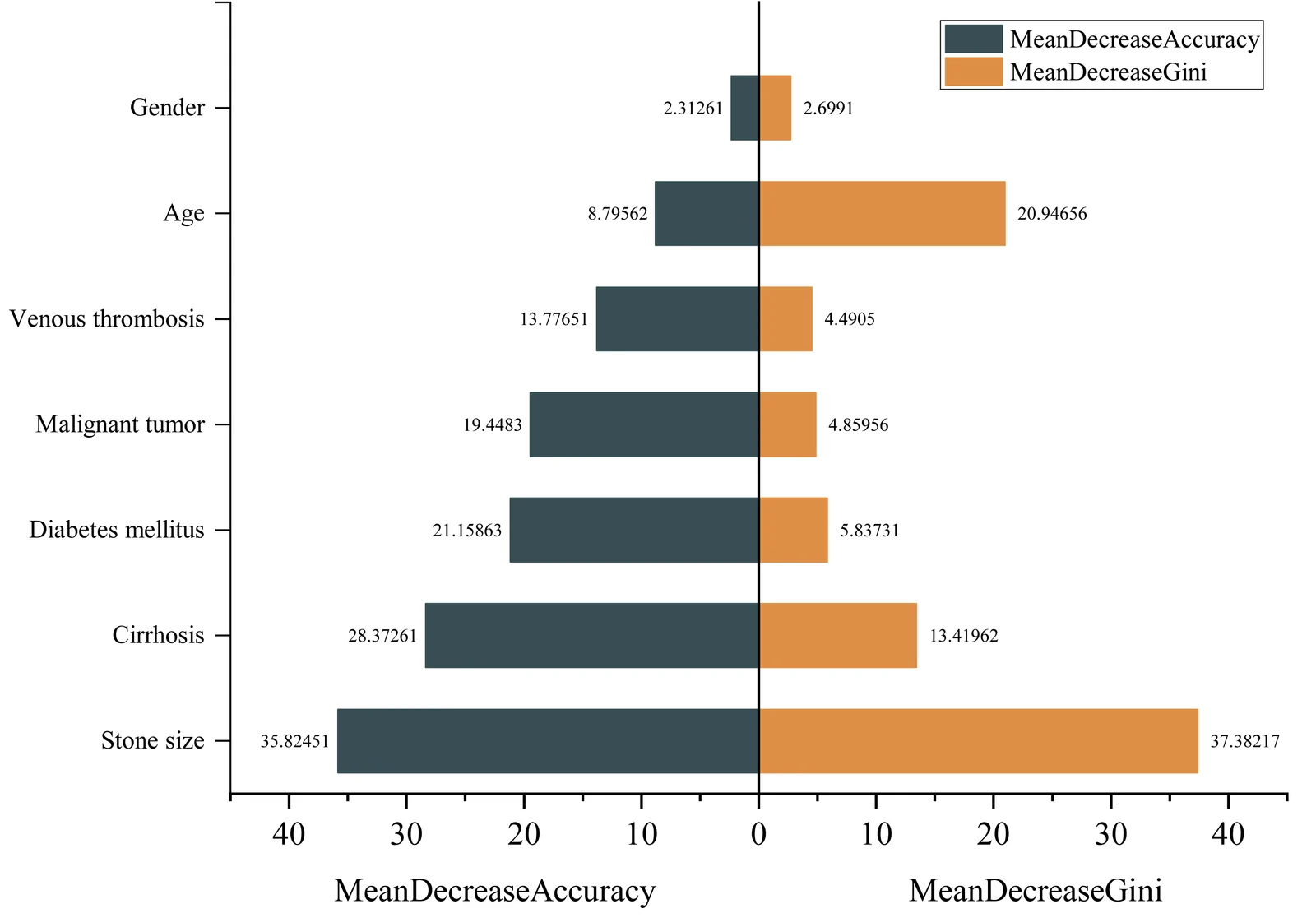
Variable importance for recurrence prediction model based on MeanDecreaseGini and MeanDecreaseAccuracy.

A Random Forest model was constructed using sex, age, and the significant predictors identified above. Variable importance analysis, based on MeanDecreaseGini and MeanDecreaseAccuracy, revealed that stone size and cirrhosis of the liver were the most influential predictors, while sex had a relatively minor impact (Fig. 5).

**Figure 6:**
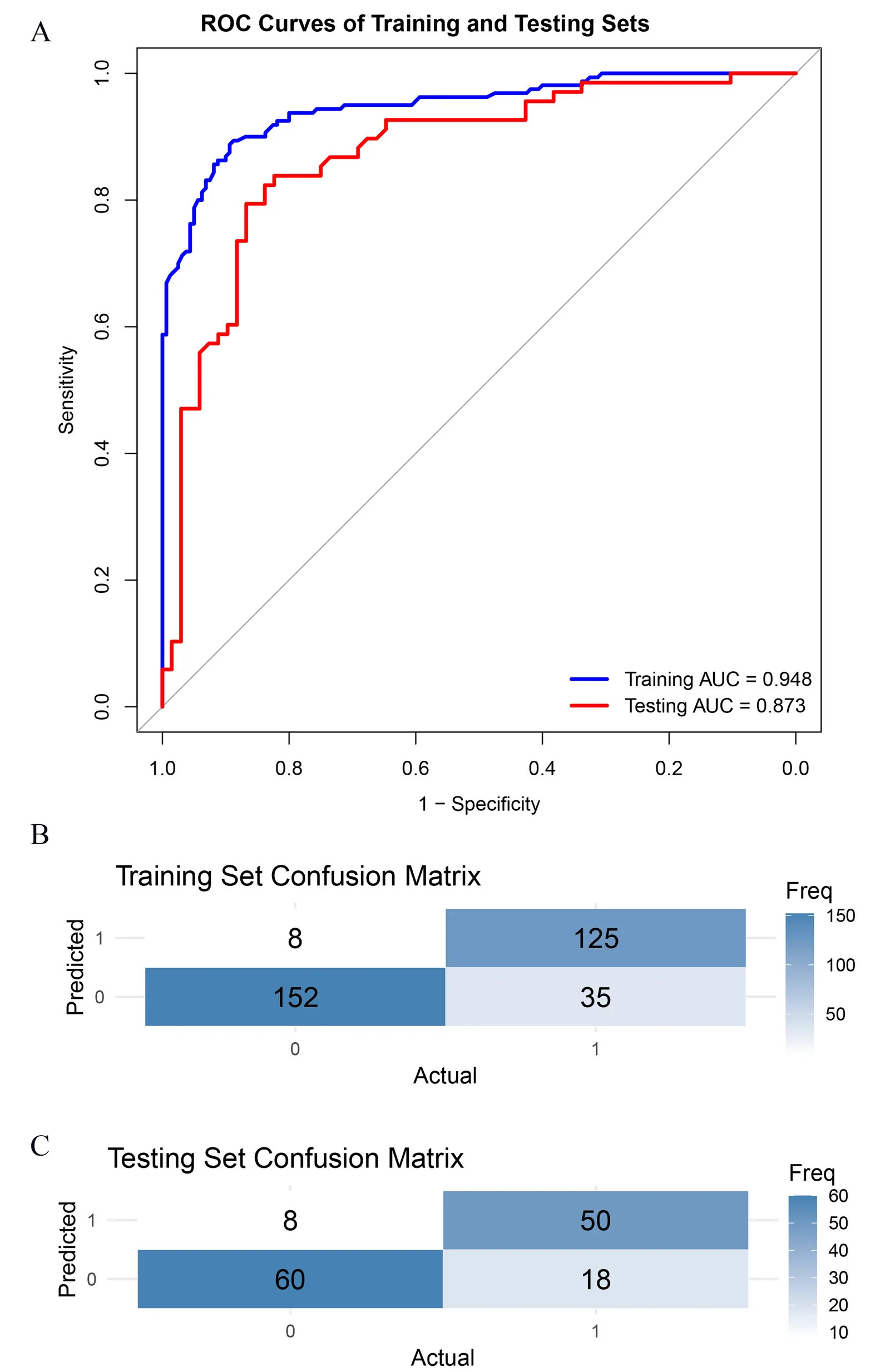
ROC curves and confusion matrices of the prediction model in the training and testing sets. (A) ROC curves of the model in the training and testing sets. The blue line represents the training set (AUC = 0.948), and the red line represents the testing set (AUC = 0.873). (B) Confusion matrix of the model in the training set. (C) Confusion matrix of the model in the testing set.

To further evaluate model performance, the model was assessed in both the training and testing sets. Receiver operating characteristic (ROC) curve analysis demonstrated excellent discriminative ability, with an area under the curve (AUC) of 0.948 in the training set and 0.873 in the testing set (Fig. 6A). Although the performance was higher in the training set, the model maintained good discrimination in the testing set, suggesting stable predictive performance without evidence of substantial overfitting. Confusion matrices for the training and testing sets are presented in Figs. 6B and 6C, further illustrating stable classification performance across datasets. Detailed performance metrics are summarized in Table 2. Overall, the model showed strong predictive performance for postoperative gallstone recurrence risk stratification.

## Discussion

This study, through an analysis of a cohort of 9,939 patients undergoing gallstone surgery, delineated clinical heterogeneity of the disease and constructed a machine learning model for predicting postoperative recurrence (AUC = 0.873). Consistent with the overall research approach outlined in the Introduction, our work moves beyond traditional anatomical classification to characterize distinct clinical phenotypes and their associated recurrence risks. Our findings suggest conceptualizing gallstone disease as a “clinical syndrome” driven by distinct pathophysiological mechanisms (Lammert et al., 2016; Patel & Jepsen, 2024), rather than as a homogeneous disease entity. We found that stone distribution exhibits significant sex and age preferences: females are more prone to cholecystolithiasis, while EBD stones are more common in males and older patients. More importantly, stones at different anatomical locations were associated with specific comorbidity networks, suggesting distinct underlying etiological niches. For instance, our analysis confirms that cholecystolithiasis is strongly associated with gallbladder polyps and adenomyomatosis, consistent with previous literature (Riddell et al., 2023). This suggests a clinical phenotype centered on local gallbladder pathology, potentially involving abnormal bile composition, chronic inflammation, or motility disorders (Lam et al., 2021). The strong association between hepatolithiasis and cirrhosis is well-established, reflecting disease progression from recurrent cholangitis to biliary cirrhosis (Fan & Wong, 1992; Mori, Sugiyama & Atomi, 2006). EBD stones emerged as a strong risk factor for recurrence, suggesting a phenotype of biliary dynamics disturbance. Prior studies have linked this phenotype to sphincter of Oddi dysfunction, duodeno-biliary reflux, and biliary dysbiosis (Liu et al., 2023; Yang et al., 2025). Previous research has also shown that certain stone morphologies, particularly small spiculated pigment stones, are more prone to migration and are associated with an increased risk of gallstone pancreatitis (Calomino et al., 2026).

**Table 2:** Performance metrics of the Random Forest model in training and testing sets.

Metrics	Training set	Testing set
Accuracy	0.87	0.81
Precision	0.78	0.74
Recall	0.94	0.86
F1-score	0.85	0.79
Sensitivity	0.94	0.86
Specificity	0.81	0.77
Youden Index	0.75	0.63

This large-scale, clinically data-driven phenotypic classification may provide a basis for exploring subgroup-specific mechanisms and therapeutic targets. In addition to the current location-based classification framework, more refined anatomical stratification of biliary tract involvement may further improve phenotypic characterization of gallstone disease. Although the present study categorized gallstone disease according to the primary anatomical distribution of stones, future prospective studies incorporating finer anatomical subclassifications, such as hilar bile duct involvement and segment-specific intrahepatic biliary distribution, may provide additional insights into recurrence patterns, disease progression, and individualized management strategies. Such anatomically integrated phenotyping frameworks may further enhance recurrence risk assessment and precision-oriented postoperative surveillance.

Regional and pathophysiological differences between Eastern and Western gallstone disease patterns warrant consideration when interpreting our findings. In Western populations, cholesterol gallstones predominate, closely associated with obesity, metabolic syndrome, and high-fat dietary patterns (Stinton, Myers & Shaffer, 2010; Wu, Song & Wu, 2025). In contrast, Eastern populations—particularly in East Asia—exhibit a higher proportion of pigment stones and hepatolithiasis, frequently linked to biliary tract infections and parasitic infestations. These differences have implications for recurrence risk (Lu et al., 2025; Tsui et al., 2011). For instance, the strong association between hepatolithiasis and cirrhosis observed in our Asian cohort may be less pronounced in Western settings where intrahepatic stones are rarer. In addition, in Western populations, hepatolithiasis is usually secondary to biliary stasis caused by extrahepatic factors (*e.g.*, common bile duct stones, postoperative or inflammatory strictures) or congenital diseases such as Caroli’s disease, rather than being driven by primary metabolic factors (García et al., 2023). Therefore, our model, derived from a Chinese cohort, may require external validation before application in Western populations.

Our findings suggest that gallstone recurrence is associated with both local and systemic factors. The five independent predictors—stone size, diabetes, venous thrombosis, liver cirrhosis, and malignant tumor—may represent interrelated pathophysiological processes. Stone size may serve as an indicator of the underlying lithogenic environment; clinical studies have shown that larger stone diameter is associated with increased recurrence risk (Wang et al., 2023; Zhang et al., 2025). Systemic factors such as diabetes and malignant tumors may contribute through metabolic and inflammatory pathways, as suggested by prior genetic and epidemiological studies (Chen et al., 2022; Lv et al., 2017; Teng et al., 2024). If confirmed, these findings would suggest that clinical management should consider both stone removal and systemic risk factor assessment.

The Random Forest model constructed in this study achieved favorable predictive performance (test set AUC = 0.873). Variable importance analysis revealed that stone size and liver cirrhosis are the two core factors for the model’s prediction of recurrence, generally consistent with the results of the multivariate regression analysis. More importantly, the key risk features identified by the model are generally consistent with the unique clinical spectra we discovered, defined by stone anatomical location: cirrhosis is significantly enriched in patients with hepatolithiasis, while EBD stones themselves are a notable risk factor for recurrence. The model’s risk profile appears to reflect both liver-related and biliary microenvironmental factors, though this interpretation requires prospective validation. Importantly, this predictive framework is based entirely on routinely available clinical variables—including stone size and common comorbidities—without reliance on bile microbiology or specialized laboratory biomarkers, highlighting its potential clinical value for future postoperative recurrence risk stratification. More broadly, the present study benefits from a large surgical cohort and provides a systematic characterization of clinical heterogeneity across sex, age, stone location, and recurrence status. From a therapeutic perspective, endoscopic advances such as digital cholangioscopy using the SpyGlass system after endoscopic retrograde cholangiopancreatography offer improved stone clearance and may reduce recurrence, particularly in patients with complex bile duct stones (Manes et al., 2019; Yoo et al., 2026). However, the impact of such techniques on long-term recurrence risk in different clinical phenotypes warrants further investigation.

Several limitations warrant consideration regarding the recurrence outcome. First, recurrence was identified solely through hospital readmission records at our institution. Asymptomatic recurrences—those not causing clinical symptoms—would have been missed entirely, and patients who experienced symptomatic recurrence but sought care at other hospitals were also not captured in our dataset. Therefore, we cannot calculate a valid population-level recurrence rate from this cohort. Second, this limitation necessitated the use of a matched case-control design for the prediction model, wherein recurrence cases were compared with non-recurrence controls selected based on hospital visits. Although the Random Forest model demonstrated favorable performance under internal validation using the training and testing sets, no independent external validation cohort was available in the present study. Furthermore, because the prediction model was developed using a matched case-control dataset, the recurrence proportion in the modeling dataset did not reflect the true event prevalence; therefore, calibration analysis was not performed. Future prospective multicenter cohort studies with complete follow-up are needed to externally validate the model and comprehensively evaluate both its discrimination and calibration. Although we integrated a set of clinical variables, we did not include deeper information such as diet, genetics, and stone composition (Di Ciaula et al., 2019; Rebholz, Krawczyk & Lammert, 2018). Future research should focus on: (1) Conducting prospective validation and assessing the clinical utility of the model; (2) integrating multi-omics data including genomics, metabolomics, and metagenomics to build more powerful biomarker-driven predictive systems (Hsu & Schnabl, 2023; Tang et al., 2022; Zheng, Allington & Vilarinho, 2022); (3) ultimately, based on precise risk stratification, designing interventional clinical trials to verify whether personalized management strategies targeting specific high-risk subtypes can effectively reduce recurrence rates, achieving the ultimate goal of moving from “prediction” to “prevention”.

## Conclusions

In conclusion, this large-scale cohort study demonstrates that gallstone disease is a clinically heterogeneous condition characterized by distinct demographic, anatomical, and comorbidity-associated phenotypes. Beyond traditional stone classification, our findings support a more nuanced phenotypic framework for understanding disease complexity and recurrence risk. Using routinely available clinical variables, we identified key recurrence-associated factors and developed a machine learning-based prediction model with favorable performance under internal validation. Together, these findings provide a promising framework for future precision risk stratification and may facilitate individualized postoperative management following prospective external validation.

## Supplemental Information

10.7717/peerj.21699/supp-1 Supplemental Information 1 Schematic diagram of gallstone classification by anatomical location the three major types of gallstones based on their location in the biliary tree: cholecystolithiasis (gallbladder stones), intrahepatic bile duct stones (IBD), and extrahepatic bile duct stones (EBD).

10.7717/peerj.21699/supp-2 Supplemental Information 2 Stone size and comorbidity information (A): Distribution of stone sizes. (B): Number and proportion of comorbidities. (C): Categories and proportions of comorbidity combinations (each patient belongs to only one category).

10.7717/peerj.21699/supp-3 Supplemental Information 3 Differences in clinical characteristics among different types of gallstones (A): sex, complications, comorbidities (B): stone size. Only variables with statistically significant differences between groups are shown. The a, b, c, d above the bar chart indicates the difference significance; Different letters indicate a significant difference between two groups, while the same letter indicates no difference between two groups.

10.7717/peerj.21699/supp-4 Supplemental Information 4 Differences in clinical characteristics between patients with and without complications (A): types of gallstones, comorbidities; (B): age; (C): stone sizes. Only variables with statistically significant differences between groups are shown. ”*” indicates *P* < 0.05; ”**” indicates *P* < 0.01; ”***” indicates*P* < 0.001.

10.7717/peerj.21699/supp-5 Supplemental Information 5 Correlation heatmap of categorical variables Only significant correlations (*P* < 0.05), with gray areas indicating no significant difference. Female is the reference category for sex, and for other binary variables, the reference category is the absence of the event. For instance, the reference category for cholecystolithiasis is no gallstones, and for recurrence, it is no recurrence.

10.7717/peerj.21699/supp-6 Supplemental Information 6 Raw clinical data of the gallstone cohort (N = 9,939)

10.7717/peerj.21699/supp-7 Supplemental Information 7 Results of univariate logistic regression analysis for factors associated with gallstone recurrence

10.7717/peerj.21699/supp-8 Supplemental Information 8 Detailed Definitions of Comorbidity Categories

10.7717/peerj.21699/supp-9 Supplemental Information 9 STROBE Checklist

10.7717/peerj.21699/supp-10 Supplemental Information 10 R code for random forest model development and ROC analysis of gallstone recurrence prediction

10.7717/peerj.21699/supp-11 Supplemental Information 11 Training dataset of gallstone patients for random forest recurrence prediction model

10.7717/peerj.21699/supp-12 Supplemental Information 12 Testing dataset of gallstone patients for random forest recurrence prediction model

## Additional Information and Declarations

### Data Availability

The following information was supplied regarding data availability: The data and code are available in the Supplemental Files.
