# Supplementary material for: Clinical heterogeneity mapping and machine learning-based prediction of recurrence in gallstone disease: a retrospective cohort study of 9,939 patients
Source: PeerJ. 2026 Sep 10;14:e21699. doi: 10.7717/peerj.21699 (PMC13571604; doi:10.7717/peerj.21699)
Supplement: Supplementary file 1 [file peerj-14-21699-s001.pdf]

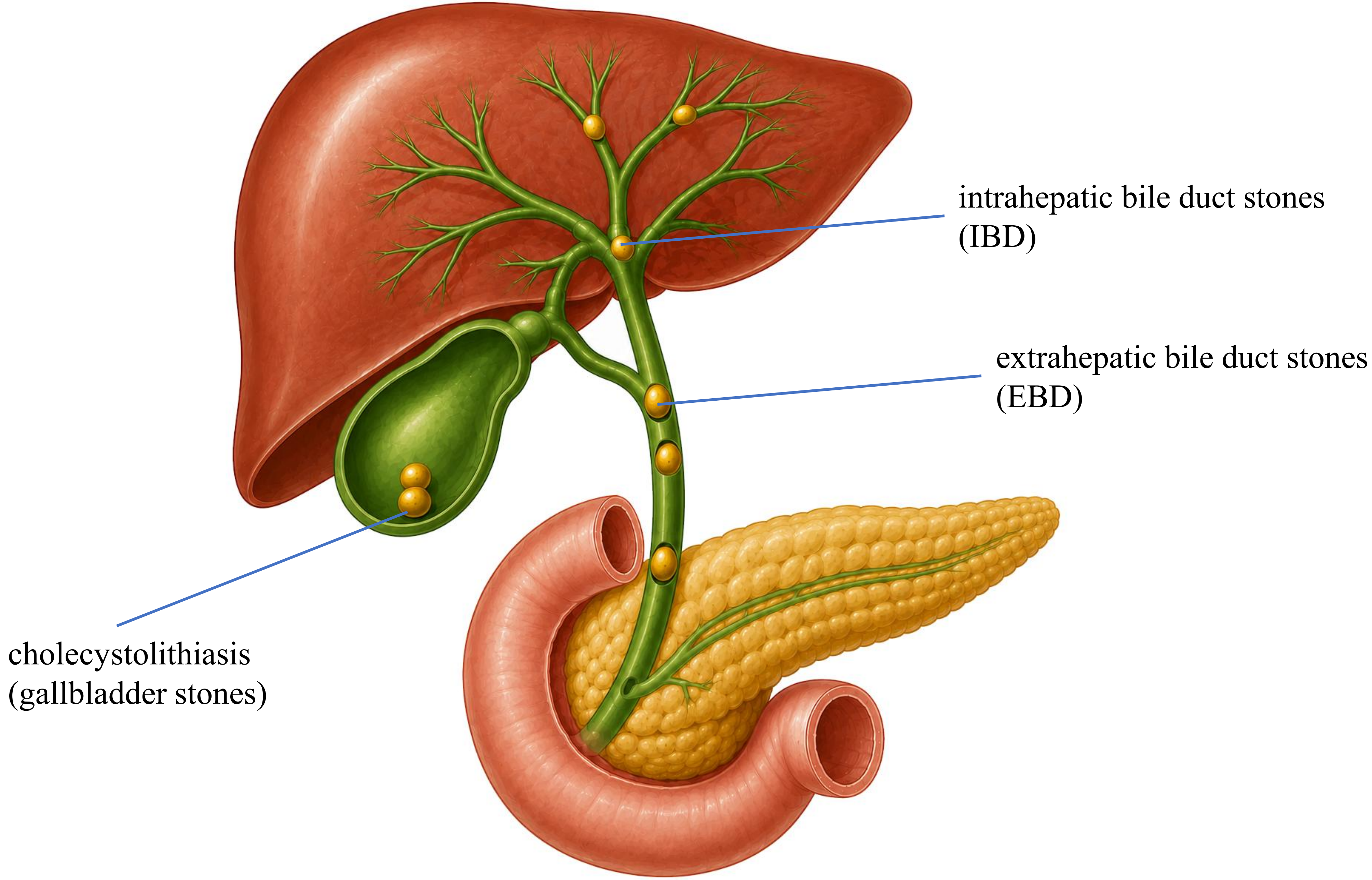
intrahepatic bile duct stones
(IBD)
extrahepatic bile duct stones
(EBD)
cholecystolithiasis
(gallbladder stones)
