# Supplementary material for: Clinical heterogeneity mapping and machine learning-based prediction of recurrence in gallstone disease: a retrospective cohort study of 9,939 patients
Source: PeerJ. 2026 Sep 10;14:e21699. doi: 10.7717/peerj.21699 (PMC13571604; doi:10.7717/peerj.21699)
Supplement: Supplementary file 2 [file peerj-14-21699-s002.pdf]

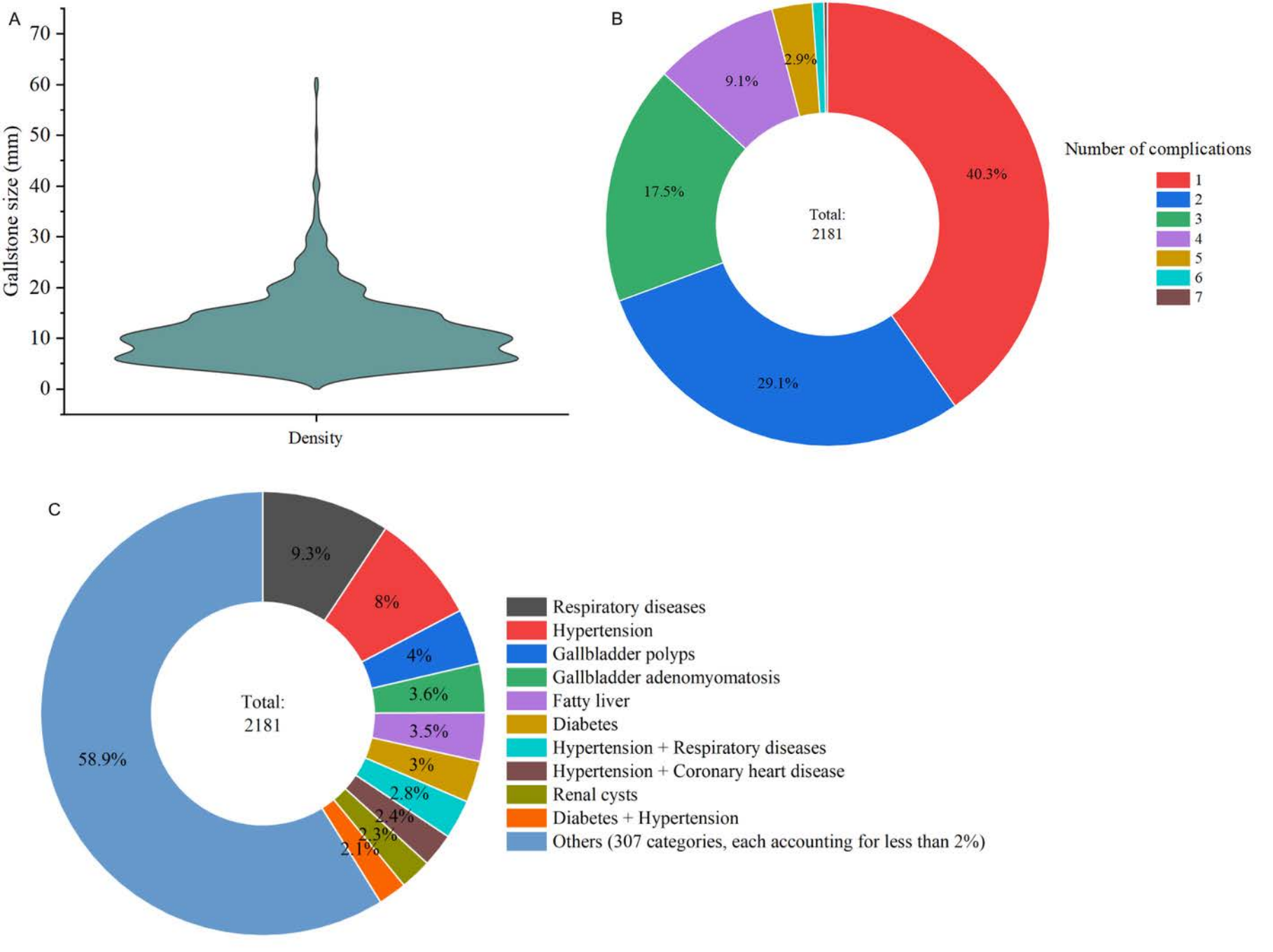
A
Gallstone size (mm)
0
10
20
30
40
50
60
70
Density
B
2.9%
9.1%
17.5%
40.3%
29.1%
Total:
2181
Number of complications
1
2
3
4
5
6
7
C
9.3%
8%
4%
3.6%
3.5%
3%
2.8%
2.4%
2.3%
2.1%
58.9%
Total:
2181
Respiratory diseases
Hypertension
Gallbladder polyps
Gallbladder adenomyomatosis
Fatty liver
Diabetes
Hypertension + Respiratory diseases
Hypertension + Coronary heart disease
Renal cysts
Diabetes + Hypertension
Others (307 categories, each accounting for less than 2%)
