# Supplementary material for: Clinical heterogeneity mapping and machine learning-based prediction of recurrence in gallstone disease: a retrospective cohort study of 9,939 patients
Source: PeerJ. 2026 Sep 10;14:e21699. doi: 10.7717/peerj.21699 (PMC13571604; doi:10.7717/peerj.21699)
Supplement: Supplementary file 3 [file peerj-14-21699-s003.pdf]

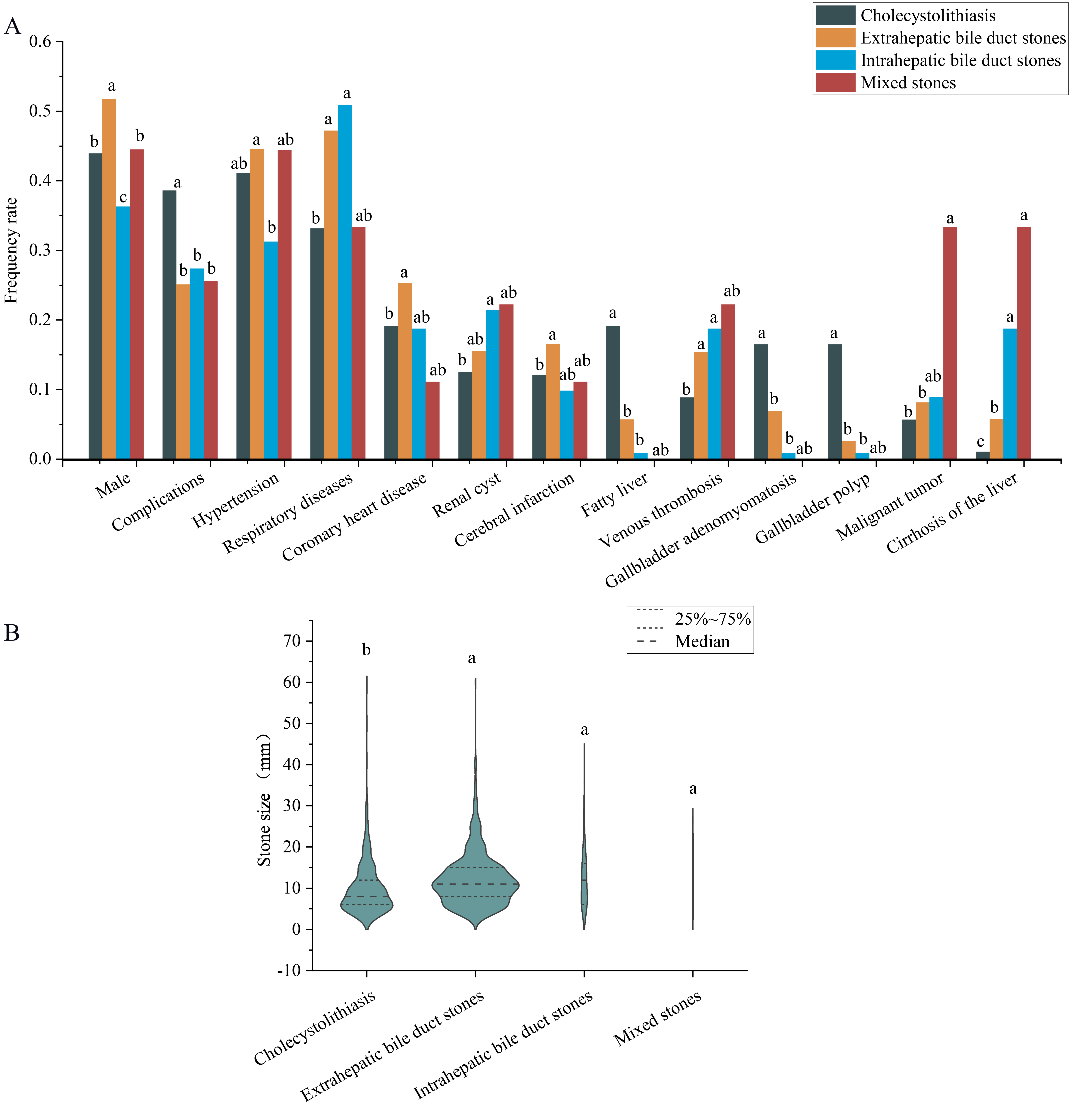

A
Cholecystolithiasis
Extrahepatic bile duct stones
Intrahepatic bile duct stones
Mixed stones
Frequency rate
0.0
0.1
0.2
0.3
0.4
0.5
0.6
Male
Complications
Hypertension
Respiratory diseases
Coronary heart disease
Renal cyst
Cerebral infarction
Fatty liver
Venous thrombosis
Gallbladder adenomyomatosis
Gallbladder polyp
Malignant tumor
Cirrhosis of the liver
B
25%~75%
Median
Stone size （mm）
-10
0
10
20
30
40
50
60
70
Cholecystolithiasis
Extrahepatic bile duct stones
Intrahepatic bile duct stones
Mixed stones
