# Supplementary material for: Clinical heterogeneity mapping and machine learning-based prediction of recurrence in gallstone disease: a retrospective cohort study of 9,939 patients
Source: PeerJ. 2026 Sep 10;14:e21699. doi: 10.7717/peerj.21699 (PMC13571604; doi:10.7717/peerj.21699)
Supplement: Supplementary file 4 [file peerj-14-21699-s004.pdf]

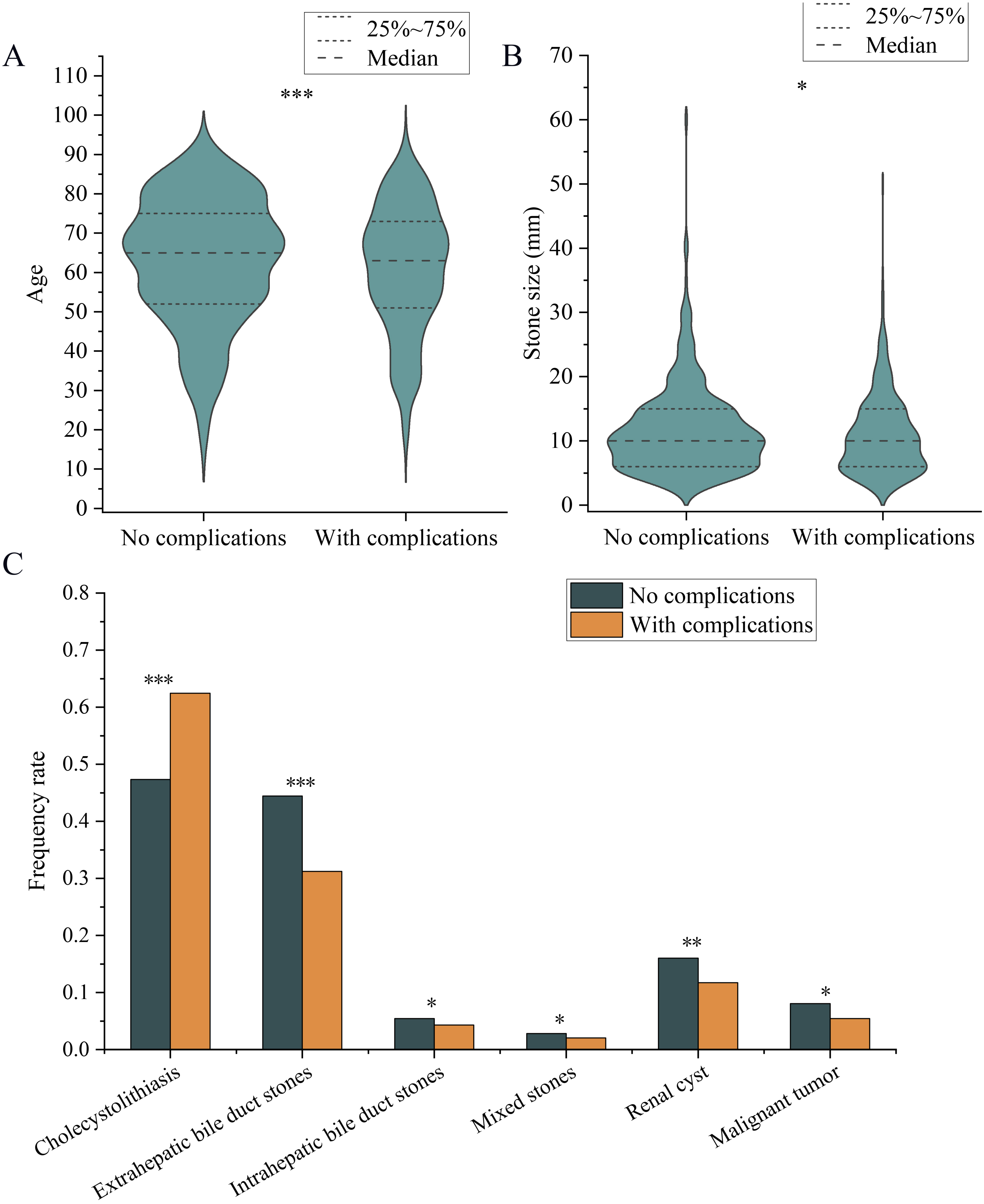
A
25%~75%
Median
***
Age
0
10
20
30
40
50
60
70
80
90
100
110
No complications
With complications
B
25%~75%
Median
*
Stone size (mm)
0
10
20
30
40
50
60
70
No complications
With complications
C
No complications
With complications
Frequency rate
0.0
0.1
0.2
0.3
0.4
0.5
0.6
0.7
0.8
***
***
*
*
**
*
Cholecystolithiasis
Extrahepatic bile duct stones
Intrahepatic bile duct stones
Mixed stones
Renal cyst
Malignant tumor
