# Supplementary material for: Clinical heterogeneity mapping and machine learning-based prediction of recurrence in gallstone disease: a retrospective cohort study of 9,939 patients
Source: PeerJ. 2026 Sep 10;14:e21699. doi: 10.7717/peerj.21699 (PMC13571604; doi:10.7717/peerj.21699)
Supplement: Supplementary file 5 [file peerj-14-21699-s005.pdf]

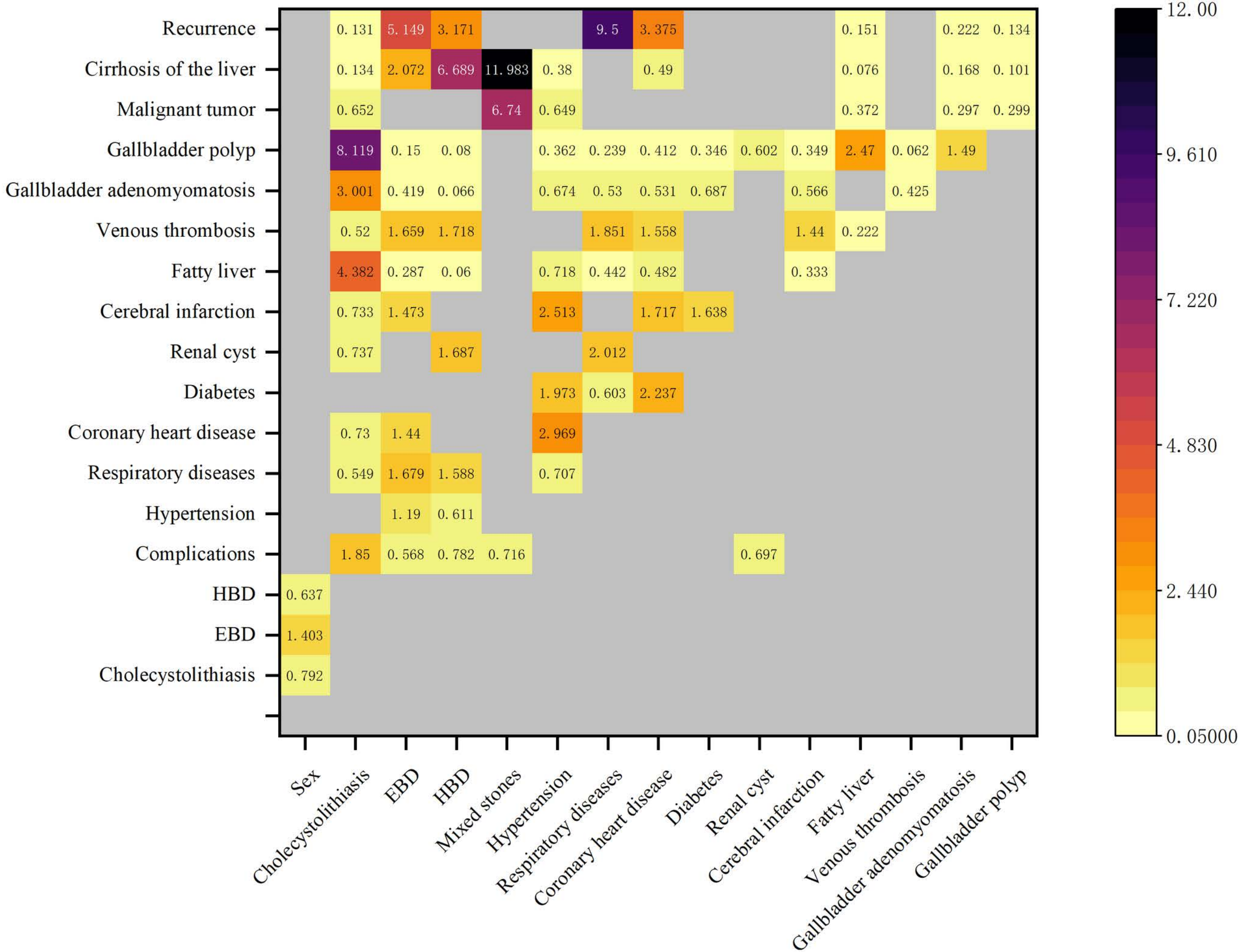
Recurrence
Cirrhosis of the liver
Malignant tumor
Gallbladder polyp
Gallbladder adenomyomatosis
Venous thrombosis
Fatty liver
Cerebral infarction
Renal cyst
Diabetes
Coronary heart disease
Respiratory diseases
Hypertension
Complications
HBD
EBD
Cholecystolithiasis
Sex
Cholecystolithiasis
EBD
HBD
Mixed stones
Hypertension
Respiratory diseases
Coronary heart disease
Diabetes
Renal cyst
Cerebral infarction
Fatty liver
Venous thrombosis
Gallbladder adenomyomatosis
Gallbladder polyp
12. 00
9. 610
7. 220
4. 830
2. 440
0. 05000
0. 131 5. 149 3. 171 9. 5 3. 375 0. 151 0. 222 0. 134
0. 134 2. 072 6. 689 11. 983 0. 38 0. 49 0. 076 0. 168 0. 101
0. 652 6. 74 0. 649 0. 372 0. 297 0. 299
8. 119 0. 15 0. 08 0. 362 0. 239 0. 412 0. 346 0. 602 0. 349 2. 47 0. 062 1. 49
3. 001 0. 419 0. 066 0. 674 0. 53 0. 531 0. 687 0. 566 0. 425
0. 52 1. 659 1. 718 1. 851 1. 558 1. 44 0. 222
4. 382 0. 287 0. 06 0. 718 0. 442 0. 482 0. 333
0. 733 1. 473 2. 513 1. 717 1. 638
0. 737 1. 687 2. 012
1. 973 0. 603 2. 237
0. 73 1. 44 2. 969
0. 549 1. 679 1. 588 0. 707
1. 19 0. 611
1. 85 0. 568 0. 782 0. 716 0. 697
0. 637
1. 403
0. 792
