# Supplementary material for: Clinical heterogeneity mapping and machine learning-based prediction of recurrence in gallstone disease: a retrospective cohort study of 9,939 patients
Source: PeerJ. 2026 Sep 10;14:e21699. doi: 10.7717/peerj.21699 (PMC13571604; doi:10.7717/peerj.21699)
Supplement: Supplementary file 6 [file peerj-14-21699-s007.docx]

**Supplementary Table S2. Results of univariate logistic regression analysis for factors associated with gallstone recurrence.**

|  | **Odds Ratio (OR)** | **95% Confidence Interval** | ***P*-value** |
| --- | --- | --- | --- |
| **Demographics** |  |  |  |
| Age (per year increase) | 0.995 | 0.979 – 1.011 | 0.504 |
| Sex (Male vs. Female) | 1.000 | 0.693 – 1.443 | 1.000 |
| **Stone Characteristics** |  |  |  |
| Maximum stone diameter (per mm increase) | 1.064 | 1.044 – 1.084 | **<0.001** |
| Gallbladder stone (Yes vs. No) | 0.424 | 0.283 – 0.635 | **<0.001** |
| Extrahepatic bile duct stone (Yes vs. No) | 1.952 | 1.323 – 2.880 | **0.001** |
| Hepatobiliary duct stone (Yes vs. No) | 2.807 | 1.320 – 5.969 | **0.007** |
| **Complications** |  |  |  |
| Cholangitis (Yes vs. No) | 0.687 | 0.375 – 1.258 | 0.224 |
| Cholecystitis (Yes vs. No) | 0.732 | 0.407 – 1.318 | 0.300 |
| Pancreatitis (Yes vs. No) | 1.000 | 0.832 – 1.202 | 1.000 |
| **Comorbidities** |  |  |  |
| Hypertension (Yes vs. No) | 1.113 | 0.769 – 1.611 | 0.572 |
| Respiratory diseases (Yes vs. No) | 2.699 | 1.839 – 3.962 | **<0.001** |
| Coronary heart disease (Yes vs. No) | 1.885 | 1.261 – 2.817 | **0.002** |
| Diabetes (Yes vs. No) | 2.063 | 1.373 – 3.100 | **<0.001** |
| Renal cyst (Yes vs. No) | 2.356 | 1.451 – 3.824 | **<0.001** |
| Cerebral infarction (Yes vs. No) | 1.000 | 0.630 – 1.586 | 1.000 |
| Fatty liver (Yes vs. No) | 1.091 | 0.612 – 1.946 | 0.768 |
| Venous thrombosis (Yes vs. No) | 3.232 | 1.991 – 5.248 | **<0.001** |
| Gallbladder polyp (Yes vs. No) | 0.908 | 0.494 – 1.669 | 0.756 |
| Gallbladder adenomyomatosis (Yes vs. No) | 0.524 | 0.205 – 1.336 | 0.177 |
| Liver cirrhosis (Yes vs. No) | 17.417 | 6.857 – 44.242 | **<0.001** |
| Malignant tumor (Yes vs. No) | 3.155 | 1.661 – 5.994 | **<0.001** |
