# Supplementary material for: Clinical heterogeneity mapping and machine learning-based prediction of recurrence in gallstone disease: a retrospective cohort study of 9,939 patients
Source: PeerJ. 2026 Sep 10;14:e21699. doi: 10.7717/peerj.21699 (PMC13571604; doi:10.7717/peerj.21699)
Supplement: Supplementary file 7 [file peerj-14-21699-s008.docx]

**Supplementary Methods**

Detailed Definitions of Comorbidity Categories

1. Respiratory Diseases

The category “Respiratory diseases” in this study encompassed the following specific conditions:

Bronchitis

Bronchiectasis

Bronchial cyst

Bronchial fistula

Bronchial asthma

Bronchial obstruction

Solitary pulmonary nodule

Emphysema

Pneumonia

Tuberculosis

Atelectasis

Pulmonary hypertension

Chronic obstructive pulmonary disease (COPD)

Pulmonary fibrosis

Pulmonary bullae

Hamartoma

Pulmonary embolism

Interstitial lung disease

Hilar lymphadenopathy

Pulmonary edema

Lung consolidation

Polycystic lung disease

Pulmonary cavity

2. Malignant Tumors

The category “Malignant tumors” in this study included the following specific malignancies:

Thyroid malignancy

Esophageal malignancy

Gastric cardia malignancy

Lung malignancy

Breast malignancy

Gastric malignancy

Secondary peritoneal malignancy

Secondary abdominal wall skin malignancy

Secondary abdominal cavity malignancy

Secondary abdominal lymph node malignancy

Secondary mediastinal malignancy

Secondary spinal malignancy

Endometrial malignancy

Liver malignancy

Bile duct malignancy

Gallbladder malignancy

Bladder malignancy

Pancreatic malignancy

Colon malignancy

Rectal malignancy

Small intestine malignancy

Duodenal malignancy

Kidney malignancy

Ureteral malignancy

Secondary lymph node malignancy

Secondary bone malignancy

Prostate malignancy

Cervical malignancy

Ovarian malignancy
