# Supplementary material for: Clinical heterogeneity mapping and machine learning-based prediction of recurrence in gallstone disease: a retrospective cohort study of 9,939 patients
Source: PeerJ. 2026 Sep 10;14:e21699. doi: 10.7717/peerj.21699 (PMC13571604; doi:10.7717/peerj.21699)
Supplement: Supplementary file 8 [file peerj-14-21699-s009.docx]

STROBE Statement—checklist of items that should be included in reports of observational studies

|  | Item No. | Recommendation | Page  No. | Relevant text from manuscript |
| --- | --- | --- | --- | --- |
| **Title and abstract** | 1 | (*a*) Indicate the study’s design with a commonly used term in the title or the abstract | 1-2 | Gallstone Disease Heterogeneity and Machine Learning Prediction of Postoperative Recurrence: A Large Retrospective Cohort Study |
|  |  | (*b*) Provide in the abstract an informative and balanced summary of what was done and what was found | 1-2 | Entire Abstract |
| Introduction | | | |  |
| Background/rationale | 2 | Explain the scientific background and rationale for the investigation being reported | 2-3 | Introduction, paras 1-3 |
| Objectives | 3 | State specific objectives, including any prespecified hypotheses | 3 | Introduction, last para |
| Methods | | | |  |
| Study design | 4 | Present key elements of study design early in the paper | 3 | Methods, Study Population and Data Collection, para 1 |
| Setting | 5 | Describe the setting, locations, and relevant dates, including periods of recruitment, exposure, follow-up, and data collection | 3 | Methods, Study Population and Data Collection, para 1 |
| Participants | 6 | (*a*) *Cohort study*—Give the eligibility criteria, and the sources and methods of selection of participants. Describe methods of follow-up  *Case-control study*—Give the eligibility criteria, and the sources and methods of case ascertainment and control selection. Give the rationale for the choice of cases and controls  *Cross-sectional study*—Give the eligibility criteria, and the sources and methods of selection of participants | 3 | Methods, Study Population and Data Collection, para 1; Clinical Definitions and Phenotyping Variables, Recurrence section |
|  |  | (*b*) *Cohort study*—For matched studies, give matching criteria and number of exposed and unexposed  *Case-control study*—For matched studies, give matching criteria and the number of controls per case | 3 | Methods, full section (no matched design reported) |
| Variables | 7 | Clearly define all outcomes, exposures, predictors, potential confounders, and effect modifiers. Give diagnostic criteria, if applicable | 3-4 | Methods, Clinical Definitions and Phenotyping Variables, full section |
| Data sources/ measurement | 8* | For each variable of interest, give sources of data and details of methods of assessment (measurement). Describe comparability of assessment methods if there is more than one group | 3-4 | Methods, Study Population and Data Collection, para 1; Clinical Definitions and Phenotyping Variables, full section |
| Bias | 9 | Describe any efforts to address potential sources of bias | 4 | Methods, Missing Data Imputation, para 1; Analysis of Recurrence Factors and Prediction Model Construction, para 2; Clinical Definitions and Phenotyping Variables, Recurrence section |
| Study size | 10 | Explain how the study size was arrived at | 3 | Methods, Study Population and Data Collection, para 1 |

Continued on next page

| Quantitative variables | 11 | Explain how quantitative variables were handled in the analyses. If applicable, describe which groupings were chosen and why | 4 | Methods, Statistical Analysis, para 1; Results, Age-Stratified Analysis section |
| --- | --- | --- | --- | --- |
| Statistical methods | 12 | (*a*) Describe all statistical methods, including those used to control for confounding | 4 | Methods, Statistical Analysis, full section; Analysis of Recurrence Factors and Prediction Model Construction, para 1 |
|  |  | (*b*) Describe any methods used to examine subgroups and interactions | 4 | Methods, Statistical Analysis, para 2; Results, Group Differences in Clinical Characteristics, full section |
|  |  | (*c*) Explain how missing data were addressed | 4 | Methods, Missing Data Imputation, full section |
|  |  | (*d*) *Cohort study*—If applicable, explain how loss to follow-up was addressed  *Case-control study*—If applicable, explain how matching of cases and controls was addressed  *Cross-sectional study*—If applicable, describe analytical methods taking account of sampling strategy | 4 | Discussion, Limitations section (para 1); no specific follow-up loss method reported in Methods |
|  |  | (*e*) Describe any sensitivity analyses | 4 | Methods, full section (no sensitivity analyses reported) |
| Results | | | | |
| Participants | 13* | (a) Report numbers of individuals at each stage of study—eg numbers potentially eligible, examined for eligibility, confirmed eligible, included in the study, completing follow-up, and analysed | 5 | Results, Baseline Characteristics of the Gallstone Cohort, para 1; Table 1 |
|  |  | (b) Give reasons for non-participation at each stage | 5 | Results, full section (no non-participation reasons reported) |
|  |  | (c) Consider use of a flow diagram | 5 | Results, Study design flowchart (Figure 1) |
| Descriptive data | 14* | (a) Give characteristics of study participants (eg demographic, clinical, social) and information on exposures and potential confounders | 5-6 | Results, Baseline Characteristics of the Gallstone Cohort, full section; Table 1 |
|  |  | (b) Indicate number of participants with missing data for each variable of interest | 5 | Results, Baseline Characteristics of the Gallstone Cohort, para 2; Methods, Missing Data Imputation, para 1 |
|  |  | (c) *Cohort study*—Summarise follow-up time (eg, average and total amount) | 5 | Results, Baseline Characteristics of the Gallstone Cohort, para 3 |
| Outcome data | 15* | *Cohort study*—Report numbers of outcome events or summary measures over time | 5-7 | Results, Baseline Characteristics of the Gallstone Cohort, para 3; Recurrence vs. Non-Recurrence section; Figure 6 |
|  |  | *Case-control study—*Report numbers in each exposure category, or summary measures of exposure |  |  |
|  |  | *Cross-sectional study—*Report numbers of outcome events or summary measures |  |  |
| Main results | 16 | (*a*) Give unadjusted estimates and, if applicable, confounder-adjusted estimates and their precision (eg, 95% confidence interval). Make clear which confounders were adjusted for and why they were included | 7 | Results, Factors Influencing Gallstone Recurrence, full section; Figure 8; Methods, Analysis of Recurrence Factors and Prediction Model Construction, para 1 |
|  |  | (*b*) Report category boundaries when continuous variables were categorized | 5-6 | Results, Age-Stratified Analysis section; Baseline Characteristics of the Gallstone Cohort, para 2 |
|  |  | (*c*) If relevant, consider translating estimates of relative risk into absolute risk for a meaningful time period | 7 | Results, Factors Influencing Gallstone Recurrence, para 2 (no absolute risk translation reported) |

Continued on next page

| Other analyses | 17 | Report other analyses done—eg analyses of subgroups and interactions, and sensitivity analyses | 5-8 | Results, Group Differences in Clinical Characteristics; Correlation Analysis Among Clinical Variables; Development and Performance of a Recurrence Prediction Model, full sections |
| --- | --- | --- | --- | --- |
| Discussion | | | | |
| Key results | 18 | Summarise key results with reference to study objectives | 8-9 | Discussion, para 1; paras 2-4 (key results linked to study objectives) |
| Limitations | 19 | Discuss limitations of the study, taking into account sources of potential bias or imprecision. Discuss both direction and magnitude of any potential bias | 10 | Discussion, Limitations section, para 1 |
| Interpretation | 20 | Give a cautious overall interpretation of results considering objectives, limitations, multiplicity of analyses, results from similar studies, and other relevant evidence | 8-10 | Discussion, paras 1-5 (full section) |
| Generalisability | 21 | Discuss the generalisability (external validity) of the study results | 10 | Discussion, Limitations section, para 1; Conclusions section, para 1 |
| Other information | |  | | |
| Funding | 22 | Give the source of funding and the role of the funders for the present study and, if applicable, for the original study on which the present article is based | 11 | Additional Information and Declarations, Funding section; Grant Disclosures section |

*Give information separately for cases and controls in case-control studies and, if applicable, for exposed and unexposed groups in cohort and cross-sectional studies.

**Note:** An Explanation and Elaboration article discusses each checklist item and gives methodological background and published examples of transparent reporting. The STROBE checklist is best used in conjunction with this article (freely available on the Web sites of PLoS Medicine at http://www.plosmedicine.org/, Annals of Internal Medicine at http://www.annals.org/, and Epidemiology at http://www.epidem.com/). Information on the STROBE Initiative is available at www.strobe-statement.org.
